# Rheological and Thermal Properties of Salecan/Sanxan Composite Hydrogels for Food and Biomedical Applications

**DOI:** 10.3390/gels11100839

**Published:** 2025-10-20

**Authors:** Xiusheng Zhang, Haihong Yang, Guangming Zhang, Xiaoxue Yan, Jun Han, Xuesong Cao, Yan Xu, Zhiping Fan

**Affiliations:** 1 Medical School, Shandong Xiehe University, Jinan 250109, China; 2 College of Agriculture and Biology, Liaocheng University, Liaocheng 252059, China; 3 Institute of BioPharmaceutical Research, Liaocheng University, Liaocheng 252059, China; 4 College of Business, Woosong University, Daejeon 35606, Republic of Korea

**Keywords:** Sanxan, Salecan, hydrogel, rheology, thermology

## Abstract

The rational design of advanced composite gels requires rigorous rheological analysis to decode their flow-deformation mechanisms, a prerequisite for optimizing performance in food and biomedical applications. However, systematic thermal analysis and rheological profiling of Salecan/Sanxan hydrogels remain unexplored, constituting a critical knowledge gap in this field. This study engineered Salecan/Sanxan hydrogels and systematically probed Salecan-dependent rheological and thermal properties. Through Power Law and Herschel–Bulkley model analyses, the hydrogels demonstrated composition-dependent rheological properties: yield stress (4.7–29.2 Pa), η_50_ (342.6–3011.4 mPa·s), and Arrhenius equation fitting revealed tunable activation energy (14,688.3–30,997.1 J·mol^−1^). Notably, when the gel was formulated with 3% Sanxan and 2% Salecan at a volume ratio of 1:2, its thermal-decomposition temperature rose by 9%, from 224.4 °C to 245.1 °C. Conversely, a 1:1 mixture of 2% Sanxan and 2% Salecan produced the lowest freezing point recorded (–18.3 °C), an 18% reduction compared with the control (–15.4 °C). These findings demonstrate the tunable rheological and thermal properties of Salecan/Sanxan hydrogels. By establishing that precise modulation of polymer mixing ratios can match the entire processing shear spectrum, this study not only fills a critical knowledge gap but also creates a versatile platform for designing tailor-made foods and biomedical matrices.

## 1. Introduction

Hydrogel, as a unique complex soft material, exhibits both solid-like elasticity and a high water content. Its three-dimensional network structure facilitates the rapid diffusion of the aqueous phase, placing it in an intermediate regime between fluid and solid [1]. Rheology—the study of material flow and deformation—offers critical guidance for engineering hydrogels whose solid–liquid character must be precisely tuned for applications ranging from food science to biomedicine [2,3]. Among the diverse hydrocolloids investigated, Sanxan has emerged as a research focal point due to its distinctive gelling properties, while Salecan is distinguished by its unique rheological and biochemical attributes. Their exceptional compatibility with hybrid cross-linking strategies positions them as ideal building blocks for next-generation hydrogels. Consequently, systematic investigation of the rheological and thermal behavior of Sanxan/Salecan composite hydrogels is imperative to establish a robust theoretical foundation for their expanded applications.

Sanxan (Sx) is a novel sphingan-class polysaccharide, which can form soft, elastic thermo-reversible gels upon cooling or by ionic crosslinking. Such gels, which are promising in texture design, functional food, and medicine development, stem from their dual gelation mechanism [4,5]. Meanwhile, sphingan-class polysaccharides possess a similar linear structure, characterized by a repeating backbone of glucose, glucuronic acid, and xylose or mannose residues, forming each tetrasaccharide unit [6]. Approved as a food additive by the National Health Commission of China in 2020, Sx is regarded as safe and a sustainable alternative to conventional colloids.

Salecan (Sal), a β-glucan from Agrobacterium ZX09 fermentation, is likewise an approved food ingredient. It consists of nine residues linked by β-(1→3) and α-(1→3) glycosidic bonds. The unique molecular structure endows Sal with versatile rheological and biochemical functions. Previous studies have reported that specific substituent patterns strongly influence its viscosity [7]. Furthermore, Sal can form high-viscosity solutions at low concentrations and remain stable over 5–95 °C and pH 1.0–13.0 [8]. Our previous research has demonstrated that Sal acts as an excellent rheological regulator in both protein gel and polysaccharide gel systems [9,10,11]. Sal further confers health benefits, including antioxidant activity [12], hypoglycaemic effects [13], and modulation of the gut microbiota [14].

At the molecular level, the mixture of these two polymers forms a stable network structure through hydrogen bonds between the carboxyl group (-COOH) of glucuronic acid in Sx’s main chain and the hydroxyl groups (-OH) of Sal. Meanwhile, the rhamnose side chains of Sx may engage in hydrophobic association with Sal’s glycosidic backbone via van der Waals forces. Additionally, under specific pH conditions, electrostatic attraction between Sx’s negatively charged groups and Sal’s protonated hydroxyl groups could further affect the stability of the composite gel. Notably, altering the Sx/Sal ratio could also induce microphase separation and interfacial effects due to the distinct chemical compositions and interaction strengths between the two polymers, leading to the formation of heterogeneous domains. The synergistic effects of these multiple structures significantly influence the mechanical and thermal properties of the resulting gels, ultimately enabling a tunable performance spectrum across different Sx/Sal ratios. To our knowledge, this study pioneers the investigation of Sal-Sx system’s performance in food gel matrices, with primary focus on developing rheology-modified solutions for safer swallowing while retaining material adaptability for diverse biomedical applications.

To evaluate how incorporating Sal affects the rheological and thermal properties of Sx-based hydrogels, we prepared a series of Sx/Sal composites via combined thermal and ionic cross-linking. Structural evolution, thermal stability, and viscoelastic response were probed by thermogravimetric analysis, FTIR, and dynamic rheometry. Rheological data were then fitted to selected constitutive models to reveal the underlying network dynamics. These findings broaden the utility of Sal in Sx hydrogels and establish a framework for designing next-generation formulations with tailored gelation and structure.

## 2. Results and Discussion

### 2.1. Morphological Structure Analysis

**Figure 1:**
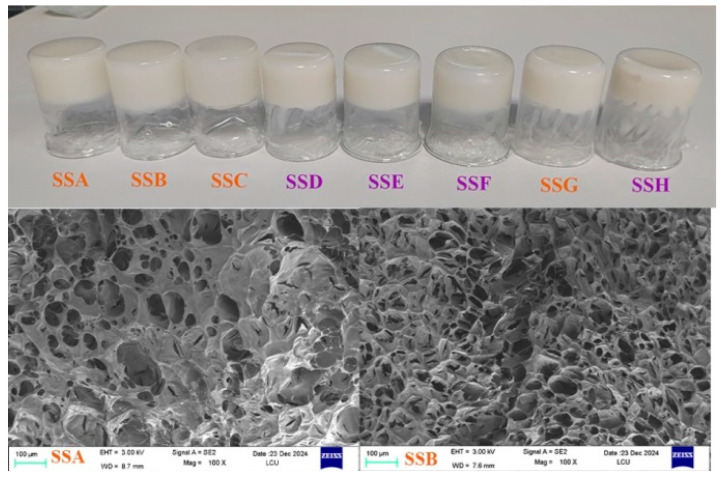

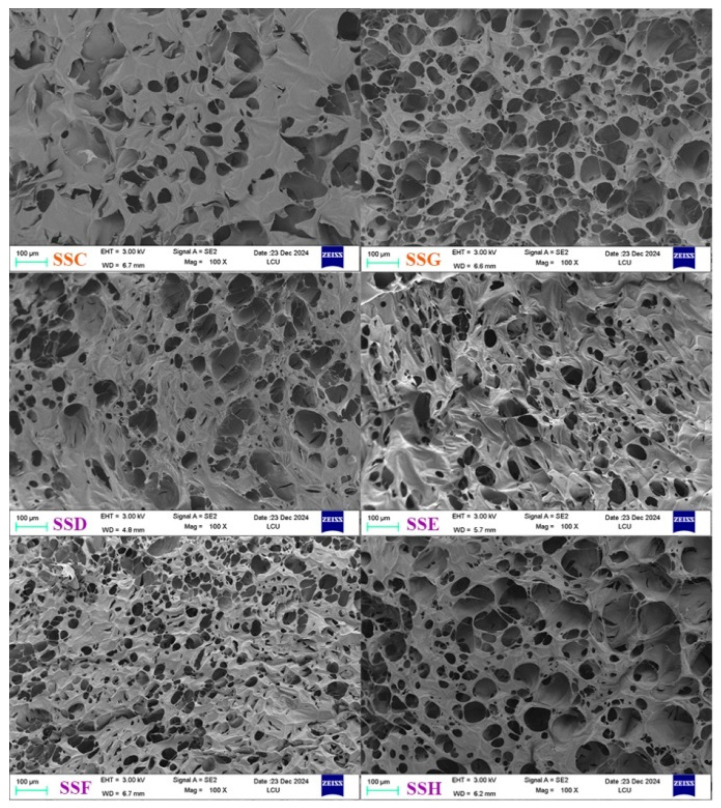
SEM image of a hydrogel sample, 100×; Average pore diameter (μm): SSA is 25.25 ± 12.18; SSB is 23.88 ± 7.82; SSC is 29.17 ± 11.32; SSD is 24.92 ± 14.01; SSE is 24.70 ± 11.39; SSF is 21.16 ± 7.40; SSG is 49.48 ± 21.04; SSH is 67.66 ± 22.84.

From Figure 1, it is observed that all hydrogel samples remain inverted in small beakers, even for those with higher Sal content (SSC), indicating that the hydrogels exhibit viscoelastic solid-state properties after formulation. Additionally, the hydrogels appear white with low transparency, a characteristic similar to that of hydrogels formed by highly acetylated gellan gum, which may be attributed to the acyl-rich conformation of the Sx polysaccharide. The SEM images in Figure 1 show that compared to pure Sx hydrogels (SSG), the composite hydrogels with added Sal exhibit smaller pore sizes, and with increasing Sal content, the pore structure becomes more uniform and refined. This presumably arises because the various substituents on the single-component Sx chains—acetyl, carboxyl and hydroxyl groups—differ in hydrophobic/hydrophilic character, thereby driving micro-phase separation (and the resultant macropores), whereas the incorporation of the miscible Sal polymer suppresses this demixing through enhanced compatibility, yielding a finer and more uniform microporous architecture. Notably, for samples with relatively high Sal content (SSC), the freeze-dried hydrogels are more prone to collapse, possibly due to reduced structural strength caused by excessive Sal addition. A comprehensive comparison of the internal microstructure reveals that the pore size of pure Sx hydrogels ranges from 20 to 110 μm, and regardless of the concentration group (2% Sx concentration: SSA, SSB, and SSC; 3% Sx concentration: SSD, SSE, and SSF), the pore size of the composite hydrogels gradually decreases to a range of 10 to 50 μm with increasing Sal content. This phenomenon highlights the role of Sal in modulating the gel network structure and reducing pore size.

### 2.2. TGA and DSC

**Figure 2:**
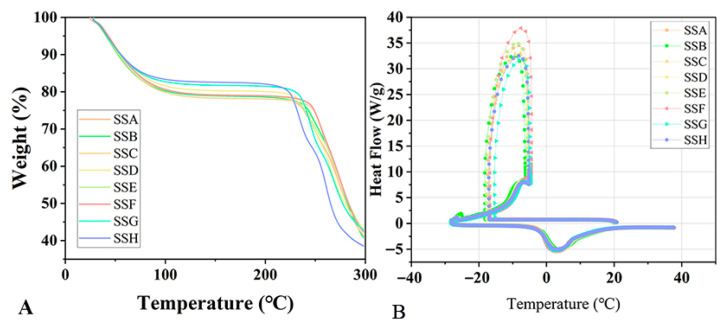
(**A**) Thermal degradation behavior of SSs; (**B**) differential scanning calorimetry of SSs.

**Figure 3:**
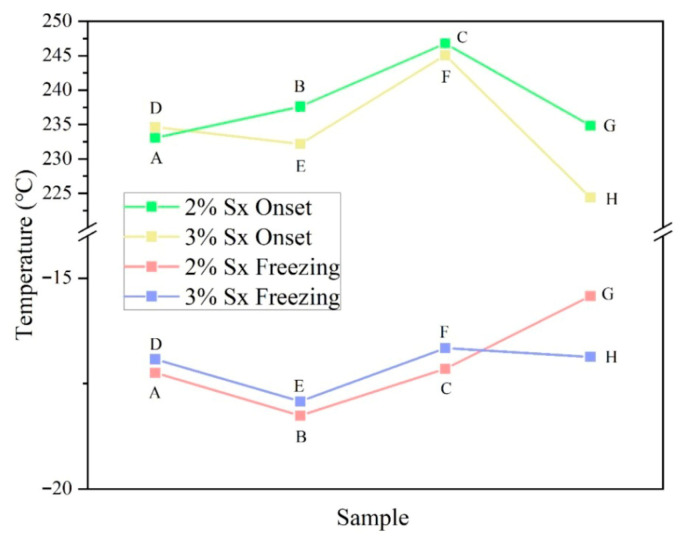
Summary of Thermal Degradation Temperature (Onset) and Freezing Point Temperature of SSs; SSA, SSB, and SSC are the 2% Sx concentration group; SSD, SSE, and SSF are the 3% Sx concentration group; SSG and SSH are control hydrogels.

The thermal stability and degradation behavior of hydrogels are often evaluated using TGA. As shown in Figure 2A, the thermal degradation curve of the freeze-dried hydrogel displays two distinct weight loss stages. The first pronounced decline (35–100 °C) corresponds to the evaporation of absorbed moisture, a characteristic behavior attributed to the material’s high hygroscopicity. This moisture uptake phenomenon occurs due to the freeze-dried hydrogel’s porous structure, which readily interacts with ambient humidity. Wu et al. also observed a similar water absorption phenomenon in Sx powder using thermogravimetric analysis [5]. The literature indicates that the polysaccharide backbone initiates thermal decomposition at approximately 230 °C [15]. When the temperature reached 220 °C, the SSs gels exhibited a second decline in the curve with further temperature increase. Studies indicated that this phenomenon is closely associated with the thermal degradation behavior of the polysaccharide backbone (such as glycosidic bonds) [16]. We present the onset degradation temperature of the SSs, as illustrated in Figure 3. It is evident that the addition of Sal to the hydrogel in both the 3% Sx (SSD, SSE, and SSF) and 2% Sx (SSA, SSB, and SSC) concentration groups follows a consistent increasing trend. The maximum increase in the onset degradation temperature of the hydrogel occurs at a Sal ratio of 1:2. This phenomenon may arise from enhanced interpolymer chain interactions (such as hydrogen bonding or electrostatic effects) or changes in microphase separation and interfacial effects. These factors synergistically improve the thermal performance of the material.

The extent to which oleophobic groups in polymers interact with water influences the thermal behavior of both the polymer and the water. For example, according to the findings of Liang’s study group, Sx reduces the amount of frozen water and enhances the flowability of salt-free noodles during the freezing process. Additionally, it attenuates the damage caused by ice crystals to the noodles and explores the mechanism through which Sx preserves the quality of salt-free noodles [17,18]. Additionally, understanding the state of water within the hydrogel—whether it is free, frozen, or unfrozen-bound water—can enhance our understanding of the hydrogel’s permeability, diffusion, and absorption properties [19].

We investigate how Sal affects the freezing point and water components of SSs using DSC. As illustrated in Figure 2B, SSs undergo two stages: an exothermic phase (cooling process) and an endothermic phase (heating process). The freezing point of SSs progressively emerges as the temperature drops. The addition of Sal lowered the freezing point of the composite gel in both the 3% Sx concentration and 2% Sx concentration groups, as shown in Figure 3. When the ratio of Sal to Sx in the hydrogel is 1:1, the freezing point of the hydrogel experiences the greatest decrease. Meanwhile, Figure 3 also indicates a consistent trend in the changes observed in the freezing point between the two groups.

Compared to the freezing point of pure free water (0 °C), the freezing point of hydrogels is significantly lower, typically ranging from −15 °C to −20 °C. This change arises from the interaction between water and polymeric materials, which alters the state of the water components and confers unique properties to the hydrogels. As high-water-content, multi-component polymeric soft materials, hydrogels contain two types of water [20,21]: free water and bound water (non-freezing bound water and freezing bound water).

The hydrogel displays a melting peak temperature (Tm), and the melting peak increases with temperature. Table 1 demonstrates that the Tm and melting enthalpy of the two groups of pure hydrogels (SSG, SSH) do not change significantly. This indicates that the water composition of the hydrogel is not substantially affected by variations in Sx concentration (2–3%). Notably, with the addition of Sal, the Tm increased for the 2% Sx concentration group. Due to the influence of hydrogen bonding, the melting temperature of freezing bound water is typically lower than that of free water [22]. This suggests that the incorporation of Sal may decrease the proportion of freezing bound water within the hydrogel, leading to an increase in its Tm temperature.

### 2.3. FTIR

**Figure 4:**
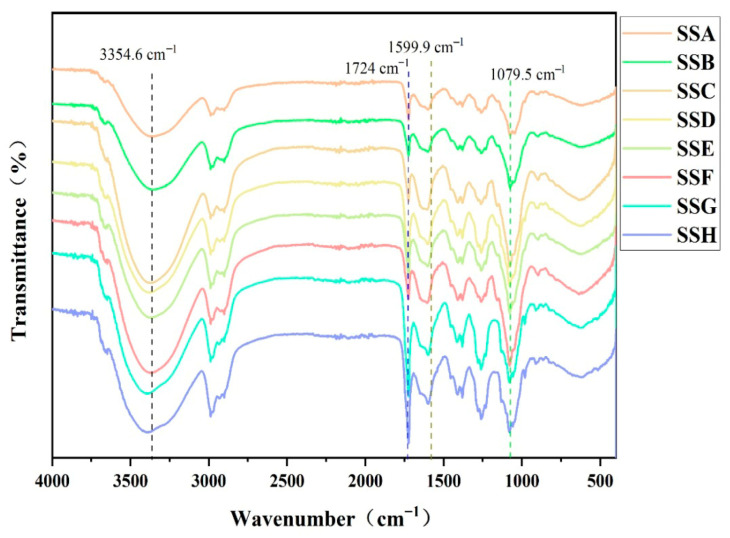
Infrared spectrum of SSs.

The primary application of FTIR is to analyze the structural changes that occur during hydrogel production [23]. A distinct absorption peak is observed at 1079.5 cm^−1^ (Figure 4), corresponding to the C-O-C glycosidic bonds [24]. Based on the observed changes in the peak of the infrared spectrum, it is evident that SSC and SSF, which have a higher proportion of Sal added, exhibit a greater absorption of C-O-C. The characteristic peak observed at 1599.9 cm^−1^ corresponds to the asymmetric stretching vibration of deprotonated carboxyl groups (COO^−^), largely consistent with previously reported FTIR signatures of calcium-crosslinked Sx hydrogels [25]. This spectral similarity suggests analogous ionic coordination between Ca^2+^ and carboxylate moieties in our system. However, unlike Lu’s [11] calcium-containing hydrogel, which showed a complete absence of protonated carboxyl (COOH) stretching at 1724 cm^−1^, our composite gel exhibited residual absorption in this region, albeit with markedly attenuated intensity upon Sal incorporation. Compared with the characteristic peak of the OH groups in the gellan pyranose ring (3307 cm^−1^) [26], the broad band at 3354.6 cm^−1^ indicates hydrogen-bond interactions occurring both intramolecularly and intermolecularly within the hydrogel, arising from –OH groups. Notably, the introduction of Sal into Sx did not result in the emergence of a new absorption peak. In summary, the Sx/Sal hydrogel prepared by thermal cross-linking and Ca^2+^-induced cross-linking exhibits ionic bonding as well as inter- and intramolecular hydrogen-bond interactions.

### 2.4. Rheology

#### 2.4.1. Discussion of Amplitude Sweep (AS)

**Figure 5:**
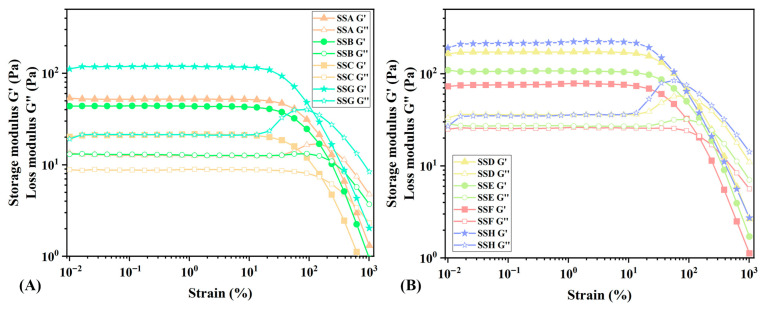
Amplitude sweeping of SSs, G ‘and G’, respectively, represent elastic modulus and viscous modulus; (**A**) represents the 2% Sx concentration group, and (**B**) the 3% Sx concentration group.

As mentioned in the introduction, it is essential to thoroughly investigate the relationship between the rheological characteristics and the physical behaviors (such as stress, strain, and temperature) of hydrogels for potential applications. Firstly, the viscoelasticity of SSs is characterized by the relationship among viscosity, elastic modulus, and large strain [27]. Dynamic amplitude scanning under varied initial flow conditions probes internal structures, with structurally intact samples enabling LVR determination for hydrogel rheological assessment [28]. Comprehending the linear viscoelastic modulus is essential for understanding network dynamics, the mesh size of gel materials, and the number of cross-linking sites [29]. The G’ of various hydrogels in the LVR is illustrated in Figure 5. Since the hydrogels in this region maintain intact, undisturbed microstructures, G’ can accurately represent the stiffness of the gel. The results revealed an inverse relationship between Sal content and hydrogel rigidity, while specimens with elevated Sx concentrations demonstrated significantly higher G’ values compared to lower-concentration counterparts. Compared with the gel stiffness (10–30 Pa) of the flaxseed arabinoxylan-modified gellan composite hydrogel reported by Mehta et al., our system demonstrates a significantly higher stiffness range (40–200 Pa) [30]. This modulus range aligns with that of soft tissues, suggesting potential applications in soft-tissue augmentation with further biomaterial development [31].

In food gel systems where mechanical properties govern texture and structural stability, multi-scale rheological analysis is particularly crucial. Consequently, testing must be conducted from small amplitude oscillatory shear (SAOS) to large amplitude oscillatory shear (LAOS). Complex fluids demonstrate various behaviors in LAOS, including strain softening, strain hardening, mild strain overshoot, and strong strain overshoot [32]. Figure 5 illustrates how the behavior of the two sets of hydrogels progressively shifts from mild strain overshoot to strain softening as the concentration of Sal increases. Notably, our study found that excessive Sal addition reduces hydrogel stiffness. Previous studies show that Sx concentrations > 0.01% form ordered microstructures, attributed to abundant acyl groups enabling side-by-side intermolecular associations as primary connections. Acyl groups further promote these associations by reducing backbone charge density, resembling high-acyl gellan gum’s ion-induced order [33]. Excessive Sal likely disrupts Sx’s intermolecular interactions and weakens its cyclic structures [5], thereby diminishing hydrogel strength.

When the shear strain exceeds LVR, the gel will yield and begin to flow as the shear strain increases. The yield point is defined as the shear stress value corresponding to the LVR boundary, representing the minimum shear stress required to disrupt the static structure of the sample and initiate flow. The flow point is defined as the shear stress value at the intersection of G’ and G” [34]. According to the data presented in Table 2, as the concentration of Sal increases, the hydrogel becomes more pliable and flows more easily. The flow transition coefficient is utilized to assess the transition behavior of the hydrogel from the LVR to the flow state. A transition coefficient approaching 1 indicates a greater tendency toward brittle fracture. As shown in Table 2, the hydrogel exhibits the most pronounced increase in flow coefficient at a 1:1 Sal-to-Sx ratio, a trend consistently observed across both 2% and 3% Sx concentration groups. By contrasting SSD with SSG, we observe that the addition of Sal increases the hydrogel’s yield stress and flow point, but does not affect its resistance to brittle fracture. This shows that the addition of Sal can enhance the resistance of Sx hydrogel to external strain. Combined with the SEM results, the addition of Sal makes the pore structure of the hydrogel more uniform and refined, but reduces the strength of the crosslinking point. Therefore, the addition of Sal does not make the Sx gel rigid or brittle, which is crucial for enhancing gel strength while maintaining resistance to brittleness. Similarly to reports on incorporating konjac glucomannan into κ-carrageenan hydrogels to reduce brittleness and improve elasticity, such combinations have been widely applied in foods like ham and jelly [35].

#### 2.4.2. Analysis of Oscillation Frequency Sweep (OFS)

**Figure 6:**
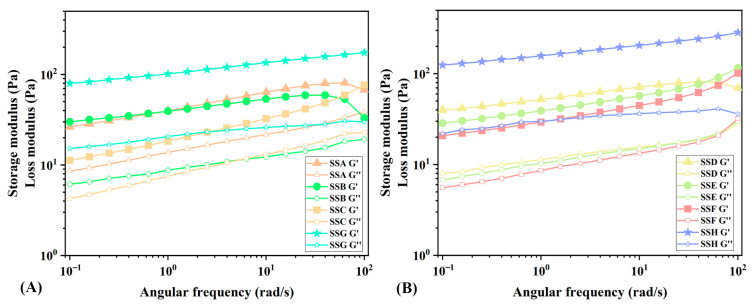
Frequency sweeping image of SSs.

Another popular technique for assessing the viscoelasticity of hydrogels is OFS [36]. Figure 6 illustrates the frequency dependence of hydrogels. The viscous and elastic modulus of the gel similarly decreases as the oscillating frequency decreases. The literature categorizes gels into three types: physical gels, chemical gels, and entangled network gels, based on their frequency-dependent behavior [37]. There is a frequency dependency in physical gels, but no crossover between G’ and G”. Chemical gel has relatively little frequency dependency and maintains a persistent covalent network. A strong frequency dependency can be observed in the G’ of the entangled network gel, which can even lead to the crossing of G’ and G”. Considering the frequency-dependent behavior and “atypical” nature of SS hydrogels, they can be described as hydrogels formed through strong physical interactions. The addition of Sal did not result in covalent crosslinking, which aligns with the findings from the infrared spectrum.

As described in the Section 4, the frequency is divided into two ranges: low frequency and high frequency. The PL equation is then utilized to fit the frequency scan curve. Table 3 demonstrates that the correlation coefficient (R^2^) is greater than 0.94, suggesting that the PL equation provides a strong fit. According to Ross Murphy, the value of b’ is zero for covalent gels and positive for physical gels [38]. The fitting results indicate that the type of SS gels is consistent with the OFS result. In addition, b’ describes the rate at which the storage modulus changes with frequency. It reflects the complexity of the internal structure of the material. By comparing the b’ value, it can be seen that the structure of the composite hydrogel shows greater complexity. It is noteworthy that SSC exhibits the highest b’ values under both high- and low-frequency oscillations. a’ and a”, respectively, represent the amplitudes of storage modulus and loss modulus at the low-frequency limit. They reflect the characteristics of materials at low frequencies. The change in stiffness of the hydrogel is quantified by a’. Whether in the 2% concentration group or the 3% concentration group, a’ decreases with the increase in the proportion of Sal added, indicating a decrease in the stiffness of the hydrogel [39]. In the composite hydrogel, SSD exhibits the highest a’ value. The elasticity index of the hydrogel is indicated by a’/a” [40]. In general, the elasticity index of SSs decreases with the increase in Sal concentration. When Sx: Sal = 1:2, the elasticity index of the hydrogel decreased the most. In the composite hydrogel, SSC exhibits the lowest elastic index, while SSB and SSD display the highest elastic indices.

Compared to the oxidized hyaluronic acid/hydrazide Sal self-healing injectable hydrogel prepared via dynamic Schiff base reaction (gel modulus: 100–300 Pa) [41], our hydrogel, prepared via physical crosslinking, exhibits a slightly lower gel modulus. However, frequency sweep results indicate that the G” of the novel composite hydrogel remains relatively stable across the entire frequency range of 100–0.1 rad/s, demonstrating its superior ability to dissipate deformation energy induced by external forces. Moreover, it exhibits higher G’ under high-frequency oscillations, a characteristic of significant importance for the storage and transportation of the hydrogel.

#### 2.4.3. Discussion of Dynamical Temperature Sweep (DTS)

**Figure 7:**
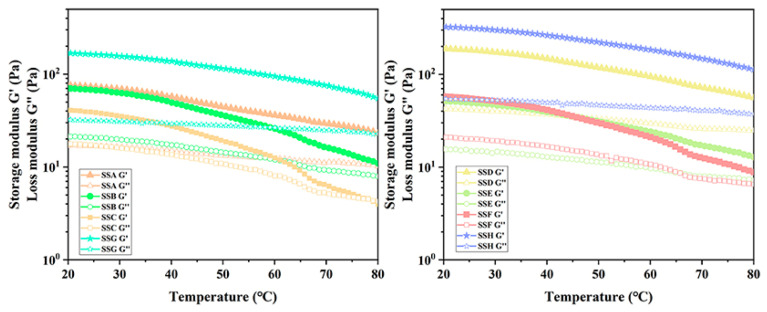
Temperature sweeping spectrum of SSs.

Gel products will experience thermal fluctuations during manufacturing, shipping, and storage. Therefore, it is essential to examine how hydrogels respond to varying temperatures. In the DTS experiment, we explore the relationship between gel properties and temperature by analyzing the functional relationship between modulus and temperature. When G’ < G”, the gel exhibited liquid-like behavior; when G’ = G”, the gel was formed; and When G’ > G”, the gel displayed solid-like characteristics [42]. Figure 7 illustrates how the viscous and elastic modulus of SSs progressively decreases as temperature increases. The G’ and G” of all hydrogels gradually decrease with increasing temperature, indicating that the intermolecular forces, such as hydrogen and ionic bonds, accelerate the dissociation as the temperature rises. Sal alters the elastic and viscous behavior of the gel, and as the temperature rises, the elastic and viscous moduli tend to converge, with G’ approaching G”. It is more logical to observe that the convergence trend of the modulus demonstrates faster convergence with an increasing fraction of Sal addition. As a clear illustration, the hydrogel (SSC) in the 2% Sx concentration group, which contains the largest fraction of Sal added, has already undergone a gel-to-sol transition when the temperature reaches 80 °C. In contrast, other hydrogels in this group did not reach G’ = G’’. These findings demonstrate that the addition of Sal does not enhance the gel-to-sol transition temperature but instead lowers it in the hydrogel.

The thermo-reversible hydrogel investigated in this study exhibits excellent stability under ambient conditions, with a gel-to-sol transition temperature exceeding 80 °C. This property ensures structural integrity during conventional storage and usage. Furthermore, the reduction in gel-to-sol transition temperature implies significantly lower energy consumption during the hydrogel processing and molding stages. This advantage is particularly critical for the industrial-scale production of hydrogels, as it not only reduces production costs but also minimizes energy consumption, thereby enhancing the environmental sustainability of the overall process. In the context of global climate change and increasing energy scarcity, the development of high-efficiency and sustainable materials is becoming increasingly urgent. Consequently, this thermo-reversible hydrogel demonstrates not only significant scientific value but also promising potential for practical applications.

The introduction of Sal may alter hydrogen bonding strength and intermolecular interactions in Sx, while also affecting the kinetic binding/dissociation rates of ionic bonds between Sx and calcium ions, thereby influencing the performance of the polymer network. To further study the thermodynamic properties of SS hydrogel, the Arrhenius equation was used to fit the temperature scanning data (Table 4), with an R^2^ value greater than 0.949, indicating a good fitting effect. Ea can be considered as the apparent activation energy of flow, which should be influenced by the dissociation of hydrogen and ionic bond binding sites [43]. It is worth noting that although the gel-to-sol transition point decreases with the increase in Sal addition, the fitted Ea value is opposite to the temperature scanning trend, meaning that Ea increases with the increase in Sal addition. The opposing trends between gel-to-sol transition temperature and Ea value may suggest that Sal addition induces cooperative structural reorganization of the Sx network, where enhanced chain cooperativity and domain formation create additional energy barriers for gel-to-sol transition, rather than simply strengthening bonds. This reorganization may involve altered chain packing dynamics or the formation of new supramolecular domains that collectively increase the energy required for thermal transitions.

#### 2.4.4. Analysis of Cyclic Strain Time Sweep (CSS)

**Figure 8:**
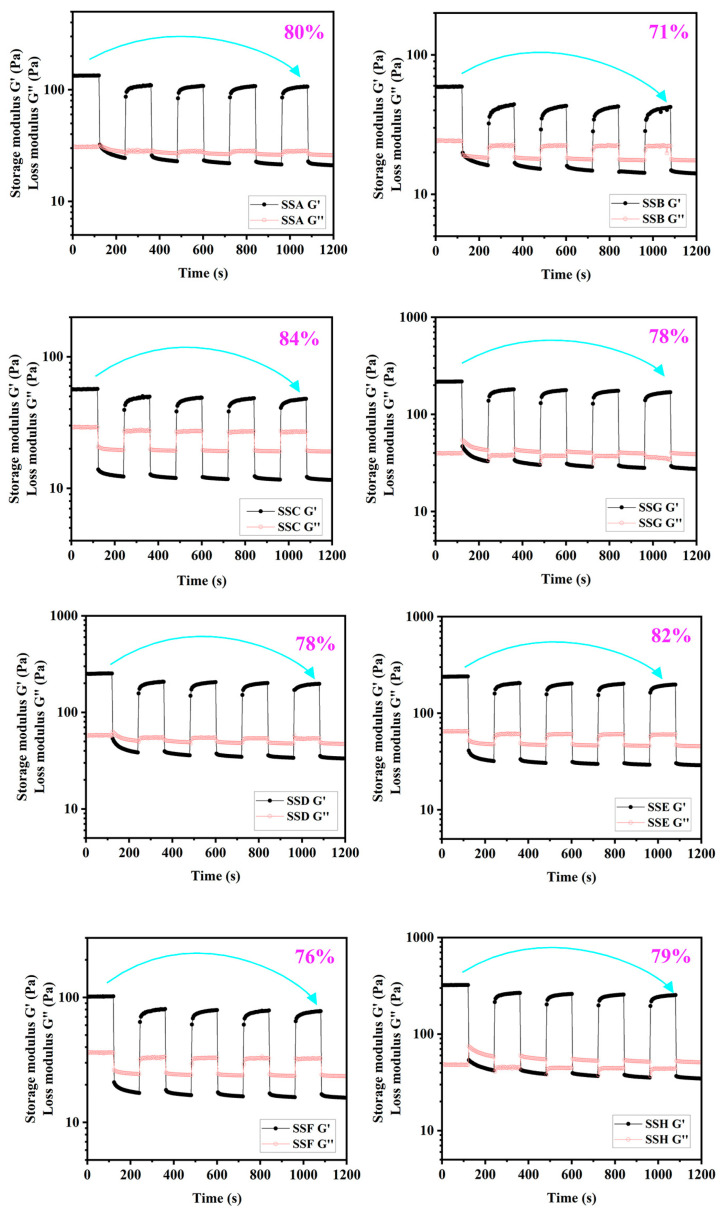
Cyclic strain time sweep diagram.

One common technique to investigate the structural deformation and regeneration of the gel under stress is to use the CSS [44]. Figure 8 illustrates that the hydrogel remains in a viscoelastic solid state after 60 s of low-strain (0.1%) application. Subsequently, the material transitions from a viscoelastic solid to a viscoelastic liquid state when subjected to a high strain (200%) beyond the flow point for an additional 60 s. The gel’s self-recovery was quantified through G’ monitoring during five consecutive cyclic deformation cycles. While the incorporation of Sal showed no significant effect on the overall recovery performance, the SSC formulation demonstrated the highest self-recovery efficiency (84%), surpassing SSG by 7.7 percentage points. In contrast to the destructive effect of konjac glucomannan on the self-recovery of gellan gels, Sal exerts virtually no adverse influence on the re-formation of the Sx hydrogel [40]. The results show that the shear stability and elastic resilience of the hydrogel are improved. Good shear stability is essential for the transportation of gel products. At the same time, good elastic resilience plays an important role in wound positioning and stabilizing the wound environment for injection of gel or shear-thinning gel. Furthermore, the G” of pure Sx hydrogel (SSG and SSH) exhibits a strain-dependent response to Sal addition: G” at high strain decreases below low-strain values, with this discrepancy amplified by higher Sal content. Lower G” correlates with reduced internal friction and bond rupture [45].

#### 2.4.5. Flow Behavior

**Figure 9:**
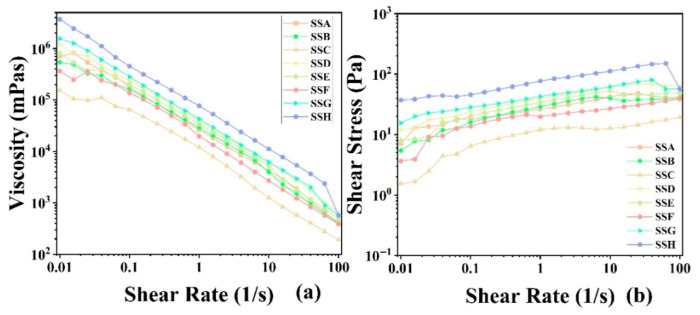
Viscosity curve (**left**) and flow curve (**right**) of SSs.

**Figure 10:**
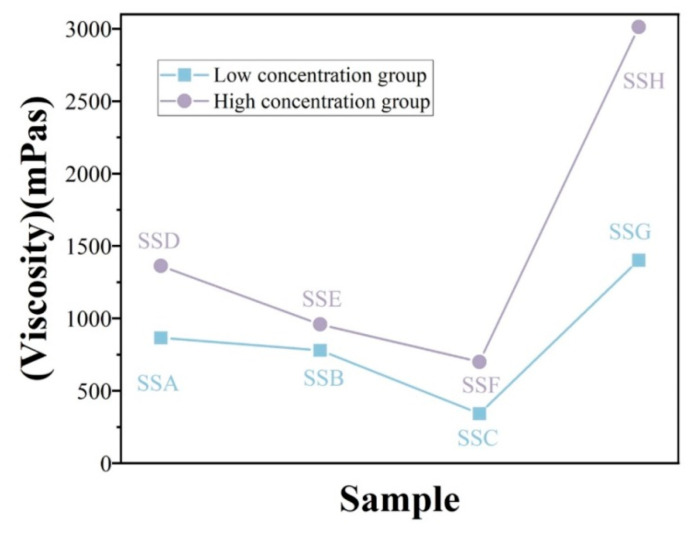
Shear viscosity (η_50_) of series hydrogels at 50 s^−1^.

The flow behavior of the hydrogel was characterised in rotational mode. Figure 9 presents the viscosity (left) and the flow (right) curves of the SSs. Upon increasing shear rate, the apparent viscosity of SSs decreased monotonically, exhibiting the characteristic shear-thinning behavior of pseudoplastic fluids [46]. Compared with flaxseed arabinoxylan-modified gellan composite hydrogels, it demonstrated more stable shear-thinning performance [30]. Similar behavior in polysaccharide–protein composites has been attributed to shear-induced disruption of hydrogen bonds and intermolecular associations [47,48]. Studies have shown that high shear-thinning behavior helps improve texture and reduce perceived viscosity, thereby facilitating swallowing [49]. We use the viscosity (η_50_) at a shear rate of 50 s^−1^ as a reference for the typical swallowing process [50]. Figure 10 demonstrates that the addition of Sal resulted in a decrease in η_50_ for both the 3% Sx concentration group and the 2% Sx concentration group. Specifically, in different concentration groups, the η_50_ of the hydrogel gradually decreased with the increasing addition of Sal. The η_50_ viscosity of all Sal-containing hydrogels ranged from 300 to 1500 mPa·s, significantly exceeding that of the xanthan gum–soy protein system (53–500 mPas) reported by Dong et al. [49]. Our hydrogel not only demonstrated higher swallowing viscosity but also exhibited superior shear-thinning behavior. These findings confirm that incorporating Sal into Sx-based gel products effectively reduces swallowing viscosity, thereby enhancing swallowability. This confirms that incorporating Sal into Sx gel products can effectively reduce swallowing viscosity, thereby improving the swallowability of the hydrogel.

Mathematical models (Power-law, PL; Herschel–Bulkley, HB) were employed to correlate shear rate (γ) with shear stress (τ), elucidate variable interdependencies, and predict flow-behavior transitions. As evidenced in Table 5, the PL model yielded the highest R^2^, indicating superior fit quality. The flow-behavior index n characterises viscosity sensitivity to shear rate [51]: n = 1 signifies Newtonian behavior, whereas n < 1 denotes shear thinning—a condition satisfied by all fitted datasets (Table 5). Among the hydrogels, SSA exhibits the lowest n value, whereas SSE shows the highest. The consistency index K, linked to zero-shear viscosity [52], monotonically decreased with increasing Sal content when derived from the PL model, mirroring the observed viscosity profiles. In the composite hydrogel, SSC exhibits the lowest K value, whereas SSD displays the highest. In contrast, the HB-derived K exhibited slight non-systematic fluctuations and reflected its additional yield-stress parameter (τ_0_).

## 3. Conclusions

In this study, a composite hydrogel composed of the gelling agent Sx and the rheology modifier Sal was fabricated. The thermal and rheological contributions of Sal to the Sx network were systematically evaluated, and the underlying molecular interactions were elucidated to predict formulation-scale utility. Scanning electron microscopy (SEM) revealed that Sal incorporation decreased pore diameter and progressively homogenized the architecture as the Sal fraction increased. TGA demonstrated that the addition of Sal increased the onset thermal degradation temperature of the composite gel, which could be attributed to enhanced interactions between polymer chains or altered crosslinking density between the components. Additionally, the DSC results reveal that the addition of Sal increased the Tm for the 2% Sx concentration group, likely due to reduced freezing bound water proportion mediated by hydrogen bonding interactions. Rheometry indicated that Sal disrupted the ring-like supramolecular associations between adjacent Sx chains, decreasing storage modulus (G’). Notably, rheological data indicated that the introduction of Sal increased the flow transition coefficient, enhanced the shear recovery rate, and decreased the gel-to-sol transition temperature of the composite gels. Collectively, Sx/Sal hydrogels exhibit ideal rheological attributes, rendering them promising candidates for dysphagia-oriented foods. It is important to acknowledge that the current study does not evaluate the long-term stability of hydrogels or their performance under practical application conditions (e.g., exposure to enzymes or physiological salts). This represents a critical limitation that must be explicitly acknowledged, as these factors are essential for real-world applicability. Future work will systematically investigate these aspects to bridge the gap between laboratory findings and clinical/environmental deployment.

## 4. Materials and Methods

### 4.1. Materials

Hebei Xinhe Biochemical Co., Ltd. (Xingtai, China) supplied the food-grade Sx (type SZ-400, 408 kDa). The batch number is 2308018. Dry weight loss of 8%, ash content of 11.2%. A quantity of Sal (C-03201201; 90% purity), with a molecular weight of 1000 kDa, was procured from Synlight Bio in Sichuan, China. The calcium chloride used in the experiment was sourced from Adamas-beta Chemical Co (Shanghai, China).

### 4.2. Hydrogel Design and Preparation

**Figure 11:**
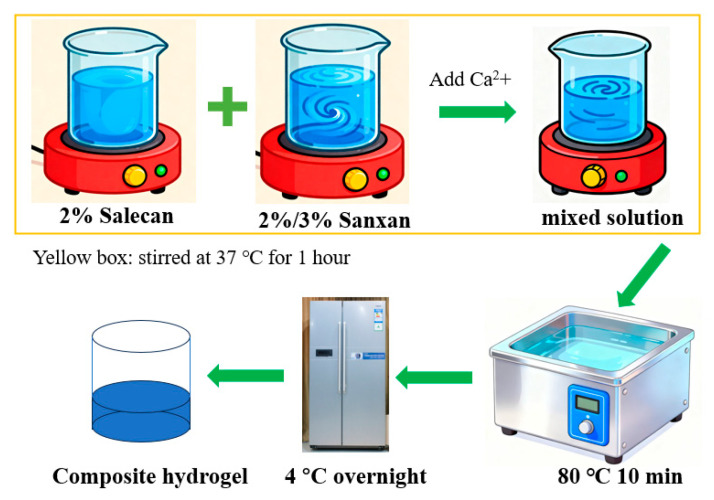
Schematic illustration of the hydrogel preparation process.

All polymer solutions were prepared using deionized water and expressed as weight/volume (*w*/*v*) percentages unless stated otherwise (Figure 11). 2% Sx: 3 g Sx was dispersed in 150 mL of water under magnetic stirring until dissolved. 3% Sx and 2% Sal solutions were prepared analogously. CaCl_2_ stock: 2.22 g CaCl_2_ was dissolved and brought to 10 mL (2 M). 2% Sx concentration series (2% Sx: 2% Sal, *v*/*v*): 2:1 (SSA), 1:1 (SSB), 1:2 (SSC). 3% Sx concentration series (3% Sx: 2% Sal, *v*/*v*): 2:1 (SSD), 1:1 (SSE), 1:2 (SSF). Control hydrogels: 2% Sx (SSG) and 3% Sx (SSH). Composite hydrogels are collectively referred to as SSs. Prepare each composite gel formulation at a total volume of 30 mL according to the above-mentioned ratio, and supplement with 0.3 mL of 2 M CaCl_2_ (final concentration 20 mM) [5]. After 10 min at 80 °C water bath, samples were transferred to 4 °C to induce gelation and stored at 4 °C until further analysis (Table 6). And, the vial tilting method revealed a consistent gelation time of ~1 h for all SSs gels in this gel environment. The comprehensive characterization included (1) Morphological analysis via scanning electron microscopy (SEM); (2) Thermal analyses using thermogravimetric analysis (TGA) and differential scanning calorimetry (DSC); (3) Chemical structural analysis by Fourier-transform infrared spectroscopy (FT-IR); and (4) Rheological assessment through amplitude sweep, oscillation frequency sweep, dynamic temperature sweep, cyclic strain time sweep, and flow behavior measurements.

### 4.3. Morphology

A digital camera (IQOO 10) was used to capture the physical appearance of the hydrogels (SSA-SSH). The internal microstructure of the hydrogels was characterized using a scanning electron microscope (SEM, GeminiSEM 300, Carl Zeiss, Berlin, Germany). The procedure involved fixing the freeze-dried hydrogels onto the SEM sample stage, followed by vacuuming and platinum sputter-coating before SEM observation. The thickness of the gold coating is 5 to 10 nanometers (nm). The accelerating voltage for SEM was set to 300 kV, and microstructural observations were conducted at magnifications of 100×. Aperture analysis was performed by randomly selecting three points from each SEM image to provide an overall characterization of the sample.

### 4.4. Thermal Analysis Characterization

The fresh hydrogel underwent a strictly controlled freeze-drying process: initially pre-frozen at −20 °C for 24 h to ensure complete water crystallization and maintain the three-dimensional network structure; followed by freeze-drying using a Virtis BenchTop Pro 31 freeze-dryer (Gardiner, New York, NY, USA) at −50 °C condenser temperature and 50 µbar vacuum pressure for 48 h to thoroughly remove bound water, ultimately obtaining a hydrogel material with intact porous structure. The freeze-dried hydrogel samples were weighed (1–5 mg) and placed into an aluminum crucible, ensuring consistent mass. Next, the crucible was placed in a thermogravimetric analyzer (TGA; TA Instruments, New Castle, DE, USA) for analysis. The temperature was increased from 25 °C to 300 °C at a rate of 10 °C/min to assess the thermal degradation behavior of the hydrogel.

To assess the freeze resistance and water-binding state of the hydrogel samples, fresh hydrogels were weighed in an open aluminum crucible, ensuring a sample mass of approximately 20 mg. To ensure consistent contact between the hydrogel and the crucible, care was taken to place the hydrogel in full contact with the crucible’s sides and bottom. The crucible was covered with an aluminum lid, and differential scanning calorimetry (DSC) was performed using a TA Instruments system (New Castle, DE, USA). The temperature program involved cycling from 20 °C to −30 °C and back to 40 °C. Nitrogen, serving as the inert gas for both thermal analyses, was used at a controlled flow rate of 50 mL/min.

### 4.5. Fourier Transform Infrared Spectroscopy

Each group’s hydrogel samples were freeze-dried, and Fourier-transform infrared (FTIR) spectroscopy, equipped with an attenuated total reflection (ATR) attachment (Nicolet iS50, Thermo Fisher, Madison, WI, USA), was employed to detect variations in functional groups among the hydrogel samples. The infrared light used for the analysis had a wavenumber range of 400 to 4000 cm^−1^.

### 4.6. Rheological Testing—Oscillation Mode

The operating modes of the rheometer include rotational and oscillatory modes. All rheological tests were conducted using parallel plate measuring equipment, with a measuring gap set at 1 mm and a plate diameter of 50 mm. The rheometer, model MCR302, was supplied by the Austrian company Anton Paar GmbH (Graz, Austria).

To obtain reliable rheological data, it is essential to employ standardized sample preloading and balancing methods. The preloading strategy involves the following steps: First, a medical spoon was used to collect a 2 mL sample of fresh gel from each group, ensuring that samples were taken from approximately the same location in the beaker. Next, the collected sample was placed in the center of the bottom plate of the measuring device. Then, the sample was ensured to fill the gap in the measuring system by lowering the top plate to the scraping position. Subsequently, any excess sample was removed using a scraper to eliminate accumulation at the edges. Finally, the system was balanced for two minutes while lowering the top plate to the measuring position.

#### 4.6.1. The Amplitude Sweep (AS)

The operating mode for shear strain control was chosen in the amplitude sweep (AS) experiment. The oscillation frequency was set to 1 Hz, and the shear strain range was set from 0.01% to 1000%. The temperature for the experiment was maintained at a constant 25 °C. According to ISO 6721-10 and EN/DIN EN 14770 standards, the tolerance range for the linear viscoelastic region (LVR) was defined using a 3% offset relative to the stable value of the storage modulus (G’). For other non-destructive oscillation tests, the strain value was typically set to 50% of the sample’s LVR value. This approach served as a preliminary validation of the test conditions before proceeding with subsequent oscillatory measurements.

#### 4.6.2. The Oscillation Frequency Sweep (OFS)

In the oscillation frequency sweep experiment (OFS), the shear strain was set to 0.1%. The angular frequency was swept from high to low, ranging from 100 to 0.1 rad/s. The experimental temperature was maintained at 25 °C. The frequencies were divided into two ranges: 0.1–1 rad/s (low frequency) and 1–100 rad/s (high frequency). The relationships between the moduli (G’ and G”) and the angular frequency were fitted using the following Equations (1) and (2) [53]:

G’ = a’ ω ^b’^ (1)

G” = a” ω ^b”^ (2)

#### 4.6.3. The Dynamical Temperature Sweep (DTS)

In the dynamical temperature sweep experiment (DTS), the applied shear strain was set to 0.1%, and the oscillation frequency was controlled at 1 Hz. The test temperature ranged from 20 to 80 °C. The edge of the parallel plate gap was sealed with silicone oil (viscosity: 0.05 Pas) to prevent water evaporation during temperature changes. The Arrhenius equation (Equation (3)), can be used to predict the relationship between soft matter and temperature.

*η* = Aexp^(Ea/RT)^ (3)

#### 4.6.4. The Cyclic Strain Time Sweep (CSS)

In the cyclic strain time sweep experiment (CSS), the sample was subjected to alternating low (0.1%) and high (200%) strains for 5 cycles (10 measurement intervals). The oscillation frequency was set at 10 Hz, and the experimental temperature was controlled at 25 °C.

### 4.7. Rheological Testing—Rotation Mode

The viscosity measured in this study is the shear viscosity, and the rheometer operates in a rotating mode to evaluate the flow behavior of soft materials. The shear rate ranges from 0.01 to 100 s^−1^. To ensure consistent flow behavior under constant temperature shear rate conditions, the test temperature was maintained at 25 °C. The data collected from the experiment were subsequently analyzed using power law equation (Equation (4)) and the Herschel–Bulkley model equation (Equation (5)).

(4) $$\tau =K\gamma^{n}$$

(5) $$\tau =\tau_{0}+K\gamma^{n}$$

### 4.8. Data Analysis

Every rotational and oscillatory test conducted in the rheological studies was repeated three times under controlled conditions. The report presents the average results of these tests. RheoCompass software 1.24 version was utilized to determine various parameters, such as shear viscosity, loss modulus, and elastic modulus. Furthermore, the collected data underwent a comprehensive fitting and analysis using Origin 2021 software and ImageJ 1.54d version.

## Figures and Tables

**Table 1:** The melting temperature and enthalpy value of SSs in DSC results.

SSs Sample	Tm (℃)	Enthalpy (J/g)
SSA	3.13	308.38
SSB	3.98	315.68
SSC	3.24	315.30
SSD	3.44	315.76
SSE	3.27	308.53
SSF	2.51	314.70
SSG	3.08	310.57
SSH	2.98	309.11

**Table 2:** Summary of yield point, flow point, and flow transition index of SSs.

SSs	Yield Point	Flow Point	Flow Transition Index
	(τ_y_, Pa)	(τ_f_, Pa)	(τ_f_/τ_y_)
SSA	11.139	47.228	4.24
SSB	5.908	35.639	6.03
SSC	4.730	15.939	3.37
SSD	22.779	97.283	4.27
SSE	14.186	65.161	4.59
SSF	10.446	43.174	4.13
SSG	15.638	67.283	4.3
SSH	29.236	90.483	3.09

**Table 3:** Summary of PL fitting parameters. The minimum (^#^) and maximum (^##^) stiffness coefficients of the SSs in the low-frequency regime; the minimum (*) and maximum (**) stiffness coefficients in the high-frequency regime. The minimum (^∆^) and maximum (^∆∆^) frequency exponents of the SSs in the low-frequency regime; the minimum (^†^) and maximum (^††^) frequency exponents of the SSs in the high-frequency regime.

Samples	G’ = a’ω^b’^			G’’ = a’’ω^b’’^		
	a’	b’	R^2^	a’’	b’’	R^2^
Low frequency (0.1–1 rad s^−1^)						
SSA	39.566	0.179	0.999	13.743	0.209	0.989
SSB	39.084	0.116 ^∆^	0.997	8.666	0.151	0.989
SSC	18.209 ^#^	0.212 ^∆∆^	0.999	7.480	0.249	0.999
SSD	51.980 ^##^	0.121	0.998	11.373	0.156	0.987
SSE	38.937	0.137	0.999	10.359	0.183	0.996
SSF	29.181	0.149	0.998	8.530	0.194	0.996
SSG	101.248	0.105	0.998	20.395	0.133	0.989
SSH	157.228	0.103	0.996	30.374	0.135	0.963
High frequency (1–100 rad s^−1^)						
SSA	42.258	0.169	0.971	12.972	0.227	0.987
SSB	40.616	0.111	0.960	8.321	0.179	0.978
SSC	15.062 *	0.338 ^††^	0.970	7.456	0.250	0.991
SSD	54.701 **	0.106 ^†^	0.946	11.242	0.143	0.974
SSE	35.952	0.211	0.973	10.063	0.178	0.984
SSF	26.711	0.235	0.975	8.264	0.213	0.983
SSG	102.595	0.116	0.998	21.609	0.076	0.968
SSH	156.4	0.119	0.998	31.004	0.066	0.973

**Table 4:** Summary of fitting parameters for Arrhenius equation.

Sample Label	lnA	Ea (J·mol^−1^)	R^2^
SSA	2.64	16,815.1	0.988
SSB	0.92	25,655.3	0.966
SSC	3.54	30,997.1	0.949
SSD	3.39	17,298.9	0.972
SSE	1.29	19,345.8	0.964
SSF	1.36	26,335.4	0.956
SSG	4.23	14,923.6	0.961
SSH	4.97	14,688.3	0.965

**Table 5:** PL and HB related fitting parameters. The minimum (^#^) and maximum (^##^) consistency index (K), and the smallest (^Δ^) and largest (^ΔΔ^) flow behavior index (n) in the Power Law model.

Sample	PL			HB			
	K (Pa·s)	n	R^2^	τ_0_ (Pa)	K (Pa·s)	n	R^2^
SSA	24.652	0.130 ^∆^	0.921	6.225	17.895	0.167	0.885
SSB	22.990	0.142	0.875	4.830	17.602	0.174	0.841
SSC	9.344 ^#^	0.159	0.873	1.003	8.160	0.177	0.853
SSD	36.613 ^##^	0.152	0.918	11.412	24.109	0.205	0.865
SSE	32.523	0.232 ^∆∆^	0.976	6.548	24.954	0.297	0.952
SSF	18.902	0.158	0.939	2.158	16.662	0.151	0.900
SSG	41.804	0.177	0.998	11.633	29.740	0.218	0.982
SSH	74.839	0.175	0.994	30.570	41.042	0.245	0.950

**Table 6:** Composition of hydrogel (SSs).

Label	Sx(*W*/*V*)	Sal(*W*/*V*)	Sx: Sal ^a^	Final Polymer Concentration Ratio (Sx:Sal)
SSA	2%	2%	2:1	1.33%:0.67%
SSB	2%	2%	1:1	1%:1%
SSC	2%	2%	1:2	0.67%:1.33%
SSD	3%	2%	2:1	2%:0.67%
SSE	3%	2%	1:1	1.5%:1%
SSF	3%	2%	1:2	1%:1.33%
SSG	2%	-	-	2%:0
SSH	3%	-	-	3%:0

^a^: Total volume 30 mL.

## Data Availability

The corresponding author will furnish requested data upon inquiry. For additional details or specific data requests, please directly contact the corresponding author. This work was prepared without the use of AI tools, and the author bears complete responsibility for the publication’s content.
